# Sex-specific differences in risk factors of lymph node metastasis in patients with early gastric cancer

**DOI:** 10.1371/journal.pone.0224019

**Published:** 2019-10-18

**Authors:** Eun Sook Ryu, Seung Jun Chang, Jungsuk An, Jun-Young Yang, Jun-Won Chung, Yoon Jae Kim, Kyoung Oh Kim, Dong Kyun Park, Kwang An Kwon, Seungyoon Nam, Woon Kee Lee, Jung Ho Kim

**Affiliations:** 1 College of Medicine, Gachon University, Incheon, Republic of Korea; 2 Department of Internal Medicine, Gachon University Gil Medical Center, Gachon University School of Medicine, Incheon, Republic of Korea; 3 Department of Pathology, Gachon University Gil Medical Center, Gachon University School of Medicine, Incheon, Republic of Korea; 4 Department of Life Sciences, Gachon Advanced Institute of Health Sciences & Technology, Gachon University, Incheon, Republic of Korea; 5 Gachon Institute of Genome Medicine and Science, Gachon University Gil Medical Center, Incheon, Republic of Korea; 6 Department of Surgery, Gachon University Gil Medical Center, Gachon University School of Medicine, Incheon, Republic of Korea; 7 Gachon Medical Research Institute, Gachon University Gil Medical Center, Incheon, Republic of Korea; National Cancer Center, JAPAN

## Abstract

Accurate prediction of lymph node status is of crucial importance in the appropriate treatment planning for patients with early gastric cancer (EGC). Some studies have examined factors predicting lymph node metastasis (LNM) in EGC; however, these studies did not consider sex-specific differences. This study aimed to investigate sex-specific differences in predictive risk factors of LNM in EGC based on surgical specimens. Patients who underwent surgical treatment for EGC between January 2003 and February 2016 were retrospectively evaluated. Patients who underwent previous gastric surgery or treatment for gastric neoplasms were excluded. Finally, 1076 patients treated for EGC were included in the analysis. We analyzed risk factors of LNM by dividing patients into male and female groups. Of 1076 patients (mean age 59.6 years), 69% were men. The overall LNM rate was 9.4%. The LNM rate was lower in men (7.8%) than in women (12.9%). Multivariate analysis showed that elevated type (odds ratio [OR], 2.084; 95% confidence interval [CI]: 1.053–4.125; P = 0.035), submucosal invasion (OR, 2.162; 95% CI: 1.018–4.595; P = 0.045), undifferentiated type (OR, 2.044; 95% CI: 1.107–3.772; P = 0.022), and lymphovascular invasion (LVI) (OR, 7.210; 95% CI: 3.835–13.554; P<0.001) were independent predictive risk factors of LNM in EGC in men. However, only submucosal invasion (OR, 8.772; 95% CI: 2.823–27.259; P<0.001) and LVI (OR, 8.877; 95% CI: 3.861–20.410; P<0.001) were independent predictive risk factors of LNM in EGC in women. Submucosal invasion and LVI were risk factors of LNM in both men and women. However, elevated and undifferentiated types were risk factors in men but not in women. Clinicians should consider these sex-specific differences with regard to individualized management.

## Introduction

Gastric cancer ranks as the fourth most common cancer and the second leading cause of cancer-related mortality worldwide. With respect to frequency, gastric cancer is the first and third most commonly occurring cancer in men and women, respectively, in the United States and South Korea. The incidence and mortality rates of gastric cancer are approximately 1.6–2.1 and 1.5–2.7 times higher in women than in men [1, 2]. In South Korea, the crude and age-standardized incidence rates per 100,000 are 2.03 and 2.40 times higher in men, respectively [1]. Thus, gastric cancer definitely has sex-specific differences with respect to incidence and mortality rates. Attributes or characteristics that contribute to the disease are called risk factors, and sex is a fixed risk factor that cannot be modified.

Early gastric cancer (EGC) is defined as gastric cancer confined to the mucosa or submucosa regardless of the presence or absence of lymph node metastasis (LNM) [3]. Endoscopic resection is widely employed as treatment for EGC in patients without LNM [4–6], and surgery is performed when LNM is anticipated. The prognosis of EGC is good with a 5-year survival rate of over 90% [3, 6–8]. Considering this good prognosis, it is very important to select an appropriate treatment modality and hence to preoperatively assess LNM status for proper treatment strategy. Therefore, many studies have been performed to identify factors that can predict LNM in EGC. These studied predictive factors can influence various diagnostic and treatment guidelines. Research into these risk factors has been ongoing and active because the therapeutic approach differs from the basic framework; nonetheless, few studies on risk factors have taken into account sex-specific differences as a fixed factor.

Various previous reports have described risk factors of LNM, including invasion depth, larger size, presence of mucosal ulceration, and undifferentiated EGC [3, 9–12]. Furthermore, some studies that analyzed the entire patient population, including men and women, have reported that women are at risk of LNM [9–11]. However, there are also studies with opposing results that suggest that female sex is not a risk factor [13, 14]. The discrepancy in these results appears to be due to the characteristics of the patients being studied. When researchers determined the risk factors of LNM in EGC, most of them analyzed the total population, including men and women. However, the number of male patients with EGC is twice the number of female patients, and the prevalence of LNM with respect to each sex is not similar. Ultimately, accurate results cannot be obtained through this manner owing to a significant difference in the proportion of the two groups that are not homogeneous to each other. To more precisely identify risk factors, it is necessary to divide these two groups and identify the risk factors in each group.

In recent years, personalized medicine has been introduced throughout the entire field. Even with the same disease, the course of the disease and the therapeutic effect differ, depending on various factors such as age, sex, and genetic characteristics. In addition, the incidence and mortality rates of gastric cancer widely vary in relation to sex, although tumor characteristics are different according to sex [15, 16]. Therefore, we thought that it was essential to separately identify predictive risk factors of LNM in patients with EGC distinguished into groups according to sex, which is a clinically fixed factor. However, no studies on risk factors of LNM have divided patients into groups of men and women. Hence, the present study aimed to elucidate on the predictive risk factors of LNM in patients with EGC in relation to sex-specific differences.

## Materials and methods

### Patients

**Fig 1:**
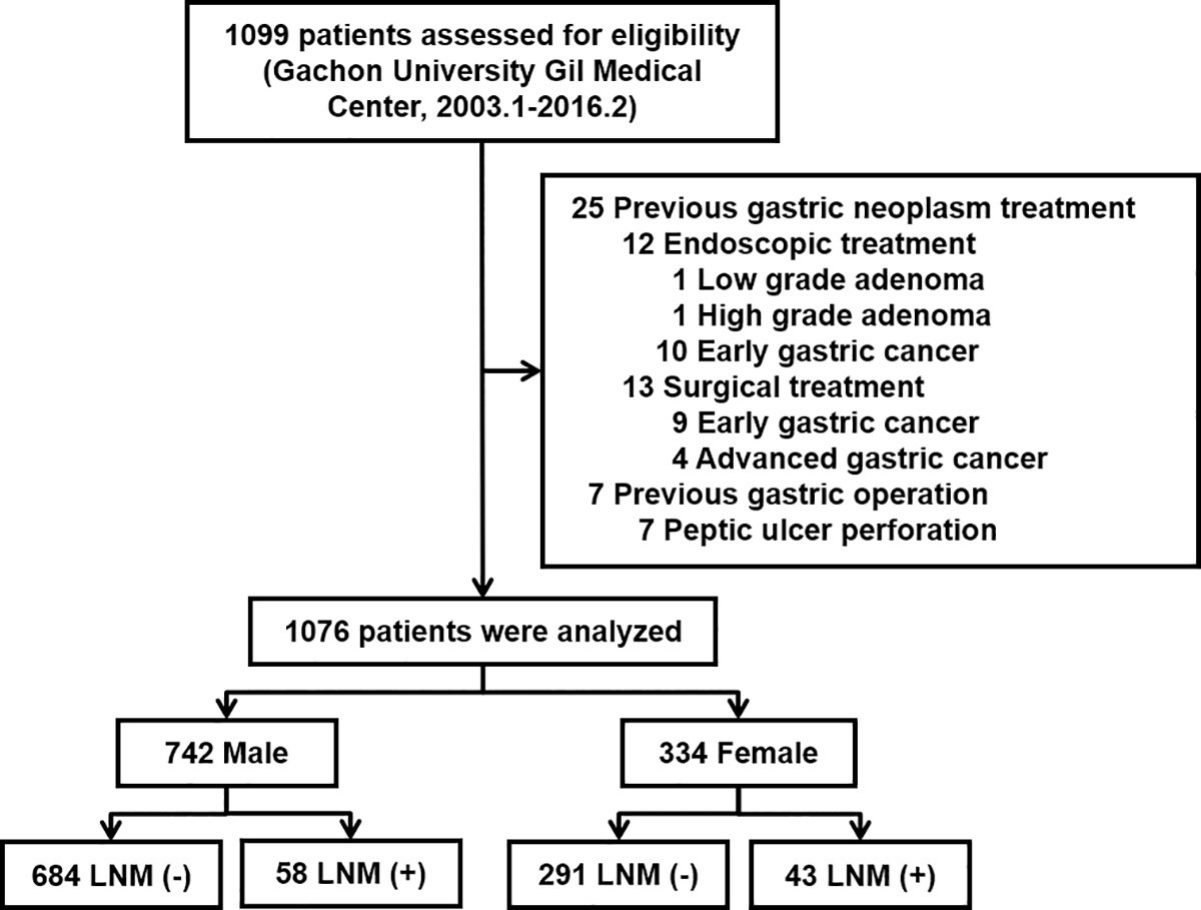
Inclusion criteria for study subjects. LNM, lymph node metastasis.

We reviewed data of 1099 patients with EGC who underwent surgical treatment at Gachon University Gil Medical Center, South Korea, from January 2003 and February 2016. Patients who underwent previous gastric surgery or received treatment for gastric neoplasm (either surgery or endoscopic treatment) were excluded from the study. Among the 1099 patients reviewed, 25 and 7 patients who received previous treatment for gastric neoplasm and underwent surgery for peptic ulcer perforation, respectively, were excluded. Of these 25 excluded patients who were treated for gastric neoplasm, 12 received endoscopic treatment, whereas 13 underwent surgical treatment (Fig 1). Finally, 1076 patients treated for EGC were included in the analysis. This study was approved by the institutional review board of Gachon University Gil Medical Center (IRB no. GAIRB2018-410) and was conducted according to the principles expressed in the Declaration of Helsinki. This study is a retrospective study using medical records, and personal information protection measures are appropriately established so that informed consent of the subject was waived.

### Definitions

As we had previously reported, we classified EGC based on location (horizontal or vertical), macroscopic findings, and histologic types in accordance with the Japanese Gastric Cancer Association criteria [17–19].

Anatomically, the stomach is equally divided into three parts based on the longitudinal axis (upper third, middle third, and lower third) and into four parts based on the vertical axis (anterior wall, greater curvature, posterior wall, and lesser curvature). The size of the EGC was measured using the maximum diameter of the lesion. EGC was macroscopically classified as either elevated, flat, or depressed and histologically categorized into two types, namely, differentiated and undifferentiated. The differentiated type included well-differentiated and moderately differentiated tubular adenocarcinomas and papillary adenocarcinomas, whereas the undifferentiated type included poorly differentiated tubular adenocarcinomas, signet-ring cell carcinomas, and mucinous adenocarcinomas.

### Statistical analysis

Categorical variables are presented as absolute numbers or percentages, whereas continuous variables are presented as means±standard deviations. In univariate analysis, categorical data were analyzed using Pearson’s chi-squared test or Fisher’s exact test. Multivariate analysis was performed using logistic regression, with the results of analysis expressed as odds ratios (ORs) with 95% confidence intervals (CIs). The patients included in the study were divided into male (n = 742) and female (n = 334) groups, and multivariate analysis was performed in each group. Using a binary logistic model, we analyzed seven probable variables—namely, age (<60 years vs. ≥60 years), tumor size (≤20 mm vs. >20 mm), vertical location (upper/middle third vs. lower third), gross type (flat/depressed type vs. elevated type), invasion depth (mucosal vs. submucosal), histology (differentiated type vs. undifferentiated type), and presence of lymphovascular invasion (no vs. yes). Statistical analysis was performed using SPSS Statistics version 22.0 (IBM Corp., Armonk, NY, USA) for Microsoft Windows. P-values <0.05 were considered statistically significant.

## Results

### Baseline characteristics

The baseline characteristics of 1076 patients are summarized in Table 1. The mean age of all patients was 59.6±12.0 years. In total, 742 (69.0%) and 334 (31.0%) patients were men and women, respectively. The mean tumor size was 30.6±19.0 mm. The tumors were most commonly located at the lower third (501, 46.6%) and lesser curvature (415, 38.6%) of the stomach in the longitudinal and horizontal axes. The most common gross appearance was the depressed type (632, 58.7%). In total, 557 (51.8%) and 519 (48.2%) patients had mucosal and submucosal invasion, respectively. The most common histological type was the moderately differentiated type (382, 35.5%). In total, 176 (16.4%) of the patients had LVI. Moreover, 196 (18.2%) and 880 (81.8%) patients underwent total gastrectomy and subtotal gastrectomy, respectively.

**Table 1:** Baseline characteristics of patients with EGC.

	Variables
Age, years	59.6±12.0
Sex	
Male	742 (69.0)
Female	334 (31.0)
Tumor size, mm	30.6±19.0
Vertical location	
UT	256 (23.8)
MT	319 (29.6)
LT	501 (46.6)
Horizontal location	
AW	233 (21.7)
GC	174 (16.2)
PW	254 (23.6)
LC	415 (38.6)
Gross type	
Elevated	161 (15.0)
Flat	283 (26.3)
Depressed	632 (58.7)
Invasion depth	
M	557 (51.8)
SM	519 (48.2)
Histology	
WD	164 (15.2)
MD	382 (35.5)
PD	199 (18.5)
SRC	325 (30.2)
Mucinous	4 (0.4)
Lymphoid stroma	2 (0.2)
LVI	
No	900 (83.6)
Yes	176 (16.4)
Operation	
T	196 (18.2)
ST	880 (81.8)

Values are presented as mean±standard deviation or n (%).

AW, anterior wall; EGC, early gastric cancer; GC, greater curvature; LC, lesser curvature; LT, lower third; LVI, lymphovascular invasion; MD, moderately differentiated adenocarcinoma; M, mucosa; MT, middle third; PD, poorly differentiated adenocarcinoma; PW, posterior wall; SRC, signet-ring cell carcinoma; SM, submucosa; ST, subtotal gastrectomy; T, total gastrectomy; UT, upper third; WD, well-differentiated adenocarcinoma.

### Risk factors of LNM in EGC according to sex

The overall LNM rate in men was 7.8% (58/742). In univariate analysis according to sex, statistically significant differences in tumor size >20 mm (87.9% vs. 68.4%; P = 0.002), elevated type (32.8% vs. 14.5%; P<0.001), submucosal invasion (81.0% vs. 47.4%; P<0.001), undifferentiated type (53.4% vs. 39.2; P = 0.033), and LVI (58.6% vs. 11.4%; P<0.001) were noted (Table 2). In multivariate analysis using logistic regression in 742 men, elevated type (OR, 2.084; 95% CI: 1.053–4.125; P = 0.035), submucosal invasion (OR, 2.162; 95% CI: 1.018–4.595; P = 0.045), undifferentiated type (OR, 2.044; 95% CI: 1.107–3.772; P = 0.022), and LVI (OR, 7.210; 95% CI: 3.835–13.554; P<0.001) were independent predictive risk factors of LNM in EGC in men (Table 2). However, age ≥60 years (OR, 1.209; 95% CI: 0.651–2.244; P = 0.548), lesion size ≤20 mm (OR, 1.906; 95% CI: 0.803–4.525; P = 0.144), and location of the lesion at the lower third of the stomach (OR, 1.418; 95% CI: 0.786–2.559; P = 0.246) were not statistically significant.

**Table 2:** Risk factors of LNM in men with EGC.

	Total (n = 742)	LNM (−) (n = 684, 92.2%)	LNM (+) (n = 58, 7.8%)	Univariate analysis	Multivariate analysis	
				P-value	OR (95% CI)	P-value
Age, years				0.636		0.548
<60	329 (44.3)	305 (44.6)	24 (41.4)		1	
≥60	413 (55.7)	379 (55.4)	34 (58.6)		1.209 (0.651–2.244)	
Tumor size, mm				0.002		0.144
≤20	223 (30.1)	216 (31.6)	7 (12.1)		1	
>20	519 (69.9)	468 (68.4)	51 (87.9)		1.906 (0.803–4.525)	
Vertical location				0.456		0.246
UT/MT	393 (53.0)	365 (53.4)	28 (48.3)		1	
LT	349 (47.0)	319 (46.6)	30 (51.7)		1.418 (0.786–2.559)	
Gross type				<0.001		0.035
F/D	624 (84.1)	585 (85.5)	39 (67.2)		1	
E	118 (15.9)	99 (14.5)	19 (32.8)		2.084 (1.053–4.125)	
Invasion depth				<0.001		0.045
M	371 (50.0)	360 (52.6)	11 (19.0)		1	
SM	371 (50.0)	324 (47.4)	47 (81.0)		2.162 (1.018–4.595)	
Histology				0.033		0.022
Differentiated	443 (59.7)	416 (60.8)	27 (46.6)		1	
Undifferentiated	299 (40.3)	268 (39.2)	31 (53.4)		2.044 (1.107–3.772)	
LVI				<0.001		<0.001
No	630 (84.9)	606 (88.6)	24 (41.4)		1	
Yes	112 (15.1)	78 (11.4)	34 (58.6)		7.210 (3.835–13.554)	

Values are presented as n (%). CI, confidence interval; D, depressed type; E, elevated type; EGC, early gastric cancer; F, flat type; LNM, lymph node metastasis; LT, lower third; LVI, lymphovascular invasion; M, mucosa; MT, middle third; OR, odds ratio; SM, submucosa; UT, upper third.

Conversely, in comparison with men, women showed the following results. The overall LNM rate in women was 12.9% (43/334). In univariate analysis, tumor size >20 mm (86.0% vs. 69.8; P = 0.027), submucosal invasion (90.7% vs. 37.5%; P<0.001), and LVI (67.4% vs. 12.0%; P<0.001) were identified as risk factors of LNM. In multivariate analysis using logistic regression in 334 women, submucosal invasion (OR, 8.772; 95% CI: 2.823–27.259; P<0.001) and LVI (OR, 8.887; 95% CI: 3.861–20.410; P<0.001) were independent predictive risk factors of LNM in EGC in women (Table 3). However, age ≥60 years (OR, 1.373; 95% CI: 0.584–3.230; P = 0.467), tumor size >20 mm (OR, 1.770; 95% CI: 0.621–5.044; P = 0.285), location of the tumor at the lower third of the stomach (OR, 1.021; 95% CI: 0.462–2.254; P = 0.959), elevated gross type (OR, 1.301; 95% CI: 0.430–3.397; P = 0.642), and undifferentiated type (OR, 2.425; 95% CI: 0.921–6.384; P = 0.073) were not statistically significant.

**Table 3:** Risk factors of LNM in women with EGC.

	Total (n = 334)	LNM (−) (n = 291, 87.1%)	LNM (+) (n = 43, 12.9%)	Univariate analysis	Multivariate analysis	
				P-value	OR (95% CI)	P-value
Age, years				0.787		0.467
<60	154 (46.1)	135 (46.4)	19 (44.2)		1	
≥60	180 (53.9)	156 (53.6)	24 (55.8)		1.373 (0.584–3.230)	
Tumor size, mm				0.027		0.285
≤20	94 (28.1)	88 (30.2)	6 (14.0)		1	
>20	240 (71.9)	203 (69.8)	37 (86.0)		1.770 (0.621–5.044)	
Vertical location				0.26		0.959
UT/MT	182 (54.5)	162 (55.7)	20 (46.5)		1	
LT	152 (45.5)	129 (44.3)	23 (53.5)		1.021 (0.462–2.254)	
Gross type				0.475		0.642
F/D	291 (87.1)	255 (87.6)	36 (83.7)		1	
E	43 (12.9)	36 (12.4)	7 (16.3)		1.301 (0.430–3.397)	
Invasion depth				<0.001		<0.001
M	186 (55.7)	182 (62.5)	4 (9.3)		1	
SM	148 (44.3)	109 (37.5)	39 (90.7)		8.772 (2.823–27.259)	
Histology				0.143		0.073
Differentiated	102 (30.5)	93 (32.0)	9 (20.9)		1	
Undifferentiated	232 (69.5)	198 (68.0)	34 (79.1)		2.425 (0.921–6.384)	
LVI				<0.001		<0.001
No	270 (80.8)	256 (88.0)	14 (32.6)		1	
Yes	64 (19.2)	35 (12.0)	29 (67.4)		8.877 (3.861–20.410)	

Values are presented as n (%). CI, confidence interval; D, depressed type; E, elevated type; EGC, early gastric cancer; F, flat type; LNM, lymph node metastasis; LT, lower third; LVI, lymphovascular invasion; M, mucosa; MT, middle third; OR, odds ratio; SM, submucosa; UT, upper third.

Subgroup analysis was performed for elevated-type or undifferentiated EGC groups with different risk factors according to sex (Table 4). Among 161 patients with elevated-type EGC, 118 patients (73.3%) were men. There were no differences in age ≥60 years, large size >20 mm, location at the lower third stomach, presence of submucosal invasion, and presence of LVI between men and women. Likewise, in patients with elevated-type EGC, there was no difference in the proportion of men and women with LVI. Additional analysis of the undifferentiated EGC group showed that 531 patients had undifferentiated EGCs, of whom 299 were men and 232 were women. Similarly, in the elevated-type EGC group, there were no statistically significant differences in age, tumor size, location, gross type, invasion depth, LVI, and LNM according to sex.

**Table 4:** Differences between elevated-type or undifferentiated EGC groups according to sex.

	Elevated EGC (n = 161)			Undifferentiated EGC (n = 531)		
	Male (n = 118)	Female (n = 43)	P-value	Male (n = 299)	Female (n = 232)	P-value
Age ≥60 years	86 (72.9)	35 (81.4)	0.269	129 (43.1)	94 (40.5)	0.543
Size >20 mm	101 (85.6)	39 (90.7)	0.395	227 (75.9)	166 (71.6)	0.255
LT location	53 (44.9)	23 (53.5)	0.335	125 (41.8)	89 (38.4)	0.422
FD type				269 (90.0)	216 (93.1)	0.202
SM invasion	80 (67.8)	27 (62.8)	0.552	146 (48.8)	97 (41.8)	0.107
Undifferentiated	30 (25.4)	16 (37.2)	0.143			
Presence of LVI	33 (28.0)	15 (34.9)	0.396	48 (16.1)	41 (17.7)	0.620
Presence of LNM	19 (16.1)	7 (16.3)	0.978	31 (10.4)	34 (14.7)	0.135

Values are presented as n (%).

D, depressed type; EGC, early gastric cancer; F, flat type; LT, lower third; LNM, lymph node metastasis; LVI, lymphovascular invasion; SM, submucosa.

### Differences in clinicopathologic characteristics according to sex

**Fig 2:**
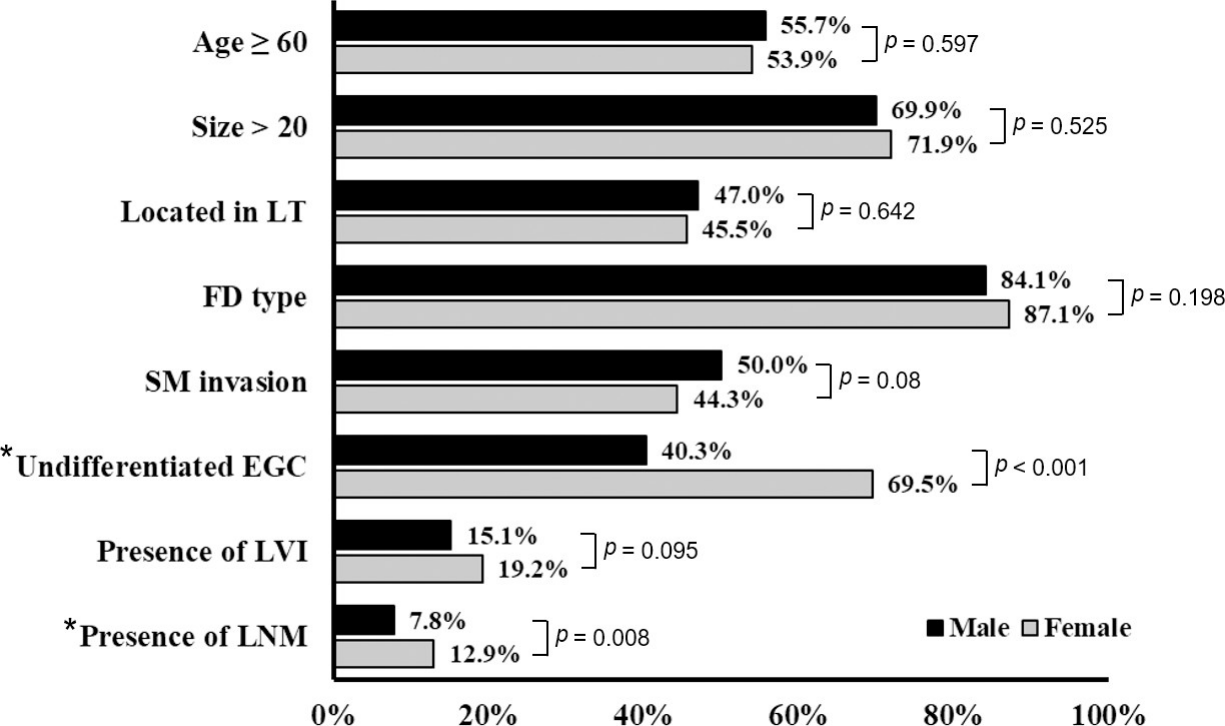
Differences in sex-specific characteristics of patients with early gastric cancer. D, depressed type; EGC, early gastric cancer; F, flat type; LT, lower third; LNM, lymph node metastasis; LVI, lymphovascular invasion; SM, submucosa.

The characteristics of male and female patients with EGC based on sex-specific differences are illustrated in Fig 2. There was no difference in the percentage of male and female patients who were ≥60 years of age (55.7% vs. 53.9%; P = 0.597); had large tumors that were >20 mm in size (69.9% vs. 71.9%; P = 0.525) and located at the lower third of the stomach (47.0% vs. 45.5%; P = 0.642); had submucosal invasion (50.0% vs. 44.3%; P = 0.08); and had LVI (15.1% VS. 19.2%; P = 0.095). However, the frequency rate of undifferentiated EGC was significantly higher in women than in men (69.5% vs. 40.3%; P<0.001); in comparison, the LNM rate was significantly higher in women than in men (12.9% vs. 7.8%; P = 0.008).

## Discussion

In our study, the overall LNM rate was 9.4%; specifically, the LNM rate was 2.7% for mucosal cancer and 16.6% (86/519) for submucosal cancer. These rates were similar to those reported by previous studies (i.e., 2%–5% for mucosal cancer and 10%–25% for submucosal cancer) [3, 20]. Interestingly, when patients were divided according to sex, the overall LNM rate was 7.8% in men and 12.9% in women. Considering the significant difference in LNM rates between men and women, this suggested that men and women with EGC were different heterogeneous groups. Based on the results of the present study, elevated type, submucosal invasion, undifferentiated type, and LVI were risk factors in men, whereas submucosal invasion and LVI were risk factors in women. In other words, elevated and undifferentiated types appeared to be risks factors in men only, not in women.

These interesting results could be attributed to two reasons. First, the molecular biology of EGC is different between men and women. The results of subgroup analysis indicated that there was no difference in clinicopathologic parameters according to sex among patients, with elevated and undifferentiated types having been identified as risk factors of LNM in men only; therefore, it should be taken into consideration that EGC in men and women may essentially show different tumor biology. Second, the distribution of clinical characteristics of EGC according to sex was different for some factors. The frequency rate of undifferentiated EGC was significantly higher in women. Owing to the high LNM rate in women, some studies reported that women are at risk for LNM [9–11, 21]. We suggest that it would be appropriate to understand that there is a sex-based difference rather than to consider female sex as risk factors and approach them individually. To consider sex-specific differences, it is necessary to consider the hormonal system to be fundamentally different depending on sex.

Unlike men, estrogen and progesterone signaling is pivotal to the establishment and maintenance of reproductive function in women. Unlike these roles, in terms of tumor biology, estrogen signaling pathway affects the initiation and development of neoplasia in breast and endometrial cancers [22]. It is possible that these hormones influence the development and progression of gastric cancer, as in breast cancer.

A previous study reported morbidity rates of 9.39%, 22.07%, and 68.54% for EGC in premenopausal female, menopausal female, and male groups, respectively, indicating a significantly higher rate in men than in women. In addition, the morbidity rate of EGC was negatively associated with estrogen level. Estrogen may provide protection against cancer [23]. Another study showed that LNM exerts an opposite effect on morbidity and reported LNM rates of 27.50%, 19.15%, and 8.90% in premenopausal female, menopausal female, and male groups, respectively. Moreover, the occurrence of LNM was positively associated with estrogen level [24]. We did not perform the present study according to hormone levels, including the presence or absence of menopause; hence, further studies on sex as a risk factor of LNM in EGC and the role of hormones in gastric cancer oncogenesis and LNM are required.

The prognosis of gastric cancer is based on TNM staging. In EGC, which is defined as gastric cancer that invades no more deeply than the submucosa irrespective of LNM, the T stage could be T1a and T1b, with any N stage. Because distant metastasis from EGC is remarkably rare (0.14%–0.37%), LNM is a very important prognostic factor and is associated with long-term survival [25, 26]. Therefore, identifying the risk factors of LNM before determining the treatment modality is very important clinically and should be approached more individually by recognizing sex-specific differences.

The present study has limitations, considering its single-institution retrospective design; nevertheless, this study is the first to investigate risk factors of LNM in EGC according to sex. EGC showed clinical and pathologic differences between men and women, and the risk factors of LNM were different owing to these differences. We hope that this study will be the starting point of research to distinguish men from women with respect to the clinical diagnosis of and treatment approach to EGC.

## Conclusions

In conclusion, invasion depth and LVI as risk factors for LNM in EGC are independent predictive factors in both men and women. However, elevated and undifferentiated types were determined to be risk factors in men only, not in women. Clinicians should consider these sex-specific differences with regard to individualized management.
